# Non-Newtonian droplet-based microfluidics logic gates

**DOI:** 10.1038/s41598-020-66337-7

**Published:** 2020-06-09

**Authors:** Elmira Asghari, Ali Moosavi, Siamak Kazemzadeh Hannani

**Affiliations:** Center of Excellence in Energy Conversion (CEEC), School of Mechanical Engineering, Sharif University of Technology, Azadi Avenue, P. O. Box 11365-9567, Tehran, Iran

**Keywords:** Engineering, Mechanical engineering

## Abstract

Droplet-based microfluidic logic gates have many applications in diagnostic assays and biosciences due to their automation and the ability to be cascaded. In spite of many bio-fluids, such as blood exhibit non-Newtonian characteristics, all the previous studies have been concerned with the Newtonian fluids. Moreover, none of the previous studies has investigated the operating regions of the logic gates. In this research, we consider a typical AND/OR logic gate with a power-law fluid. We study the effects of important parameters such as the power-law index, the droplet length, the capillary number, and the geometrical parameters of the microfluidic system on the operating regions of the system. The results indicate that AND/OR states mechanism function in opposite directions. By increasing the droplet length, the capillary number and the power-law index, the operating region of AND state increases while the operating region of OR state reduces. Increasing the channel width will decrease the operating region of AND state while it increases the operating region of OR state. For proper operation of the logic gate, it should work in both AND/OR states appropriately. By combining the operating regions of these two states, the overall operating region of the logic gate is achieved.

## Introduction

Microfluidics lab-on-chip devices have many applications in diagnostic assays, analytical chemistry, and biosciences. Using droplet-based microfluidic devices may offer various benefits^1^. One of the advantages is encapsulating the important fluids as a droplet in order to prevent them from chemical reactions and pollutions. Another advantage of these systems is a better mixing of the reactant inside the droplet and increasing the reaction speed^2^. In this manner the sample volume decreases significantly, the cost of operation is reduced drastically, and diagnostic results are obtained in a much shorter time, higher precision, sensitivity, and portability^3^.

For the droplet-based microfluidic devices, one needs to consider various components such as the valves and the mixers to perform certain functions. Electric, magnetic and thermocapillary forces are some types of forces that are used to control and manipulate fluid in microfluidic circuits^4–6^. Thus, these circuits may contain many components that will make their construction complicated. Furthermore, some external equipment such as the permanent magnet or wire coils should be considered^7–11^. Additional components limit the portability, scalability and parallel operations. The solution is automation. In electronics, complex operations are obtained using logic gates. Logic gates have many applications like sound and molecular computing^12,13^. By analogy to logic gates (in which the pressure can be analogous to the voltage, the flow rate to the electric current and the hydrodynamic resistance to the electrical resistance) one can derive their functionalities for the microfluidics^14,15^.

Fabricating fluidics devices that are similar to logic circuits, began in the 1960s^16,17^. These devices were dependent on flow inertial properties and on the high Reynolds numbers that were their main drawback; because it was not possible to shrink them in size. In miniaturized devices, the inertial force is negligible compared to the interfacial and viscous forces; thus, another source of nonlinearity is needed for the logic operation. As mentioned above, the reason is the concept of the microfluidic logic gate stems from digital logic gate and a logic circuit does not follow a straight line. This means that we want things to switch unambiguously to either ‘0’ or ‘1’, but never ‘0.5’ or other values^18^. In summary, the logic circuit in electronics are nonlinear so in microfluidics, it should be also nonlinear.

Viscoelastic polymer solutions as a working fluid were used for fluidic control, however, their operations depend on the non-Newtonian fluid properties^19,20^. Vestad *et al*. proposed a number of logic gates by controlling the relative flow resistance^21^. Since the input/output representation of these gates is not the same, these gates have not the capability of being cascaded. Cheow *et al*. and Prakash and Gershenfeld introduced bubble/droplet logic for the first time to overcome the previous problems^22,23^.

In the bubble/droplet logic using two-phase flows, the presence or absence of a bubble/droplet in the continuous phase represents one or zero, respectively. The bubble/droplet logic is based on bubble-to-bubble hydrodynamic interactions. Since the droplets have resistance, their presence in the microchannel affects the overall flow^24–27^. For example, the trajectory of the droplets entering a junction depends on the history of decisions taken by the previous droplets that are still in the outlet branches^28–33^. Under certain conditions, the droplet sequence will have a stable and repeatable pattern^34–37^. Using interactions between droplets, one could build interactive elements such as logic gates^19,20,38^, signal encoder/decoders^35–37,39,40^, sorters^41–44^, and storage units^45,46^.

Anandan *et al*. studied the previous droplet-based microfluidic logic gates numerically using the phase-field method^47^.

So far, all the bubble/droplet-based logic gates have used Newtonian fluids. No work has been done to realize how a non-Newtonian fluid flow can affect the droplet-based logic gates and in which flow characteristics the gates will operate. Since many important and practical fluids such as blood and polymer solutions are non-Newtonian, it is inevitable to study these fluids in logic microfluidics^48^.

To summarize, as explained, the lab-on-a-chip devices have many applications in diagnostic assays, analytical chemistry, and biosciences. These applications involve many parallel and serial operations. In electronics, complex operations are handled using logic gates. Analogously, in microfluidics, several logic gates have been proposed for this purpose and various experimental and theoretical studies have been conducted in this area. However, all these studies are limited to Newtonian fluids, while many practical fluids such as blood and polymer solutions are non-Newtonian. So it is inevitable to study these fluids in the logic microfluidics^48^. further, for any logic gates, there is no report to check whether a logic gate can operate properly or not in any specific conditions.

In the logic gates, many processes such as the droplet breakup, deformation or generation of droplets occur. Thus, the relation between the shear stress and the rate of the shear strain in these systems plays a critical role in such processes. In this manner, we expect that a logic gate with a non-Newtonian fluid operates different from that with a Newtonian Fluid. We will show that the logic gates that are designed for Newtonian fluids may not work with non-Newtonian fluids.

In the present study, we consider a logic gate with a power-law non-Newtonian fluid and investigate how the flow and fluid characteristics will change the logic operation. The considered system should not be very simple; because the discussion may not have the required depth and quality. And we may miss some of the necessary details. On the other hand, if it is too complicated, the main goals of the research may be affected. Therefore, a typical bubble/droplet-based is considered.

## Results and Discussion

### The governing equations and the method

Various methods have been developed and are available for modeling two-phase flows and tracking the interface between two phases^49,50^. The level set method has been extensively used for this purpose. However, the major drawback of this method is that it is not conservative. Olsson *et al*. have developed a conservative level set method to overcome this deficiency^51,52^. Furthermore, the level set method has some important advantages such as the flexibility to describe complex interface geometries, easy implementation and capturing the interface of breakup and merging automatically. In addition, the phase field method needs a large number of grids near the interface because of its rapid change in phase field function^53^. In this study, we prefer to use the level set method due to its advantages.

In this method, *ϕ* is the level set function and changes smoothly across the interface from 0 to 1 and the interface is defined where *ϕ* is 0.5. The conservative level set equation is as below:

1 $$\frac{\partial \phi }{\partial t}+\nabla \cdot (\vec{u}\phi )=\gamma \nabla \cdot \left(\varepsilon \nabla \phi -\phi (1-\phi )\frac{\nabla \phi }{|\nabla \phi |}\right)$$

where $\overrightarrow{u}$ represents the fluid velocity field, $\gamma$ is reinitialization parameter and $\varepsilon$ denotes the interfacial thickness.

In order to simulate the immiscible two-phase flow, Eq. (1) should be coupled with the Navier-Stokes and the continuity equations:

2 $$\rho \frac{\partial \vec{u}}{\partial t}+\rho (\vec{u}\cdot \nabla )\vec{u}=\nabla \cdot [-pI+\mu (\nabla \vec{u}+{(\nabla \vec{u})}^{T})]+{\overrightarrow{F}}_{st}$$

3 $$\nabla \cdot \vec{u}=0$$

where $\rho$ is density, $p$ represents the pressure, $\mu$ denotes the viscosity and ${\vec{F}}_{st}$ stands for the surface tension force. The surface tension has been introduced as a body force in the momentum equation and can be calculated from the following equation:

4 $${\vec{F}}_{st}=\sigma k\vec{n}{\delta }_{sm}$$

where $K$ is the local curvature of the interface, $\overrightarrow{n}$ represents the surface unit normal vector and ${\delta }_{sm}$ denotes the Dirac delta function and can be defined as:

5 $$k=-\,\nabla \cdot \vec{n}$$

6 $$\vec{n}=\frac{\nabla \phi }{|\nabla \phi |}$$

7 $${\delta }_{sm}=6|\phi (1-\phi )||\nabla \phi |$$

Since the droplet is a non-Newtonian power-law fluid, its viscosity is defined as below:

The shear stress tensor (${\boldsymbol{\tau }}$) and the rate of deformation tensors ($\dot{{\boldsymbol{\gamma }}}$) are related by

8 $${\boldsymbol{\tau }}=-\,\eta \,\dot{{\boldsymbol{\gamma }}}$$

The components of $\dot{{\boldsymbol{\gamma }}}$ are calculated from $\dot{{\boldsymbol{\gamma }}}=(\nabla {\bf{v}}+{(\nabla {\bf{v}})}^{\dagger })$. The effective viscosity is calculated from the magnitude of the rate of deformation tensor $\dot{\gamma }$

9 $$\dot{\gamma }=\sqrt{0.5(\dot{{\boldsymbol{\gamma }}}:\dot{{\boldsymbol{\gamma }}})}$$

where the symbol “:” represents the tensor double dot product operator. The effective viscosity can be obtained by

10 $$\mu =K\,{\dot{\gamma }}^{n-1}$$

where $n$ represents the power-law index and $K$ denotes the consistency index^54^. In the level set method, the viscosity and the density of the fluid in the entire domain are calculated from:

11 $$\mu ={\mu }_{1}+({\mu }_{2}-{\mu }_{1})\varnothing $$

12 $$\rho ={\rho }_{1}+({\rho }_{2}-{\rho }_{1})\varnothing $$

where the subscripts 1 and 2 refer to the continuous (fluid 1) and the dispersed (fluid 2) phases, respectively.

All of the above-mentioned equations were solved with the appropriate corresponding boundary conditions in a two-dimensional domain. The equations were discretized using Finite Element Method (FEM) and a Parallel Sparse Direct Solver (PARDISO) was used for solving the linear systems of equations^55^. One important issue is that at the start of the simulations the distance between the droplet from the T-junction should be large enough to allow the T-Junction area to reach undisturbed conditions of the velocity and the pressure.

#### Verification

For verification of the present study, two different droplet-based microfluidic systems with the Newtonian as well as the non-Newtonian fluid were considered. Since the droplet break up and the droplet generation play important roles in the logic gates it is essential to show that the applied method is able to model such processed correctly and precisely. The details are explained in the followings:

#### Newtonian

**Figure 1:**
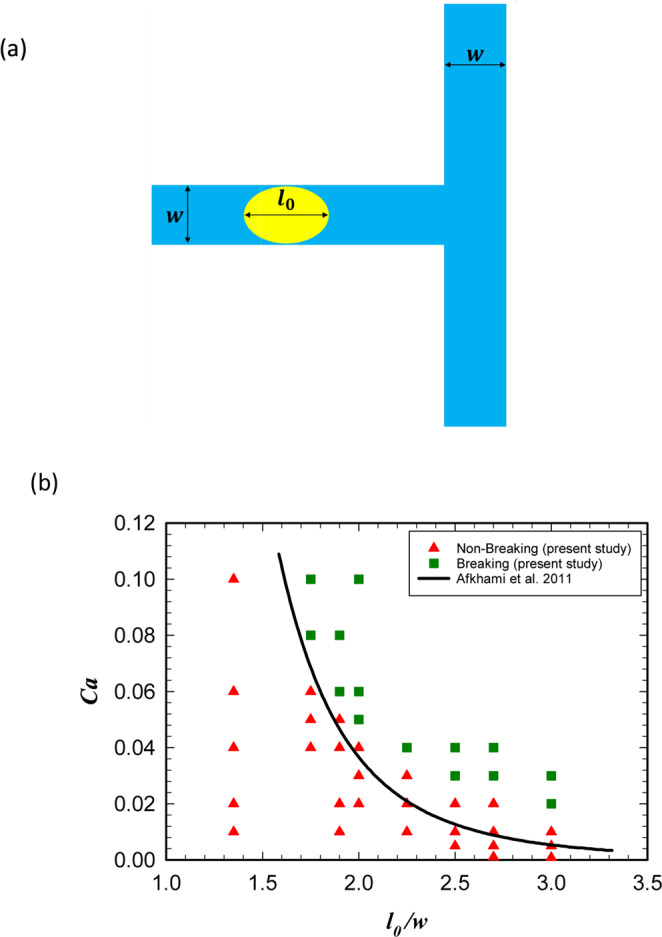
(**a**) The geometry of the microchannel used by Afkhami *et al*.^56^ for studying the breakup of droplets. (**b**) The obtained phase diagram that separates the breakup and non-break up regions. The obtained data points are exactly the same as those achieved by Afkhami *et al*.^56^.

As will be explained later in section 5.1 the droplet breakup process has been used in the considered logic gate. Therefore, to verify the model, the problem of droplet breakup in a symmetric microfluidic T-junction was considered. Afkhami *et al*. have reported a phase diagram that specifies the breakup and non-breakup regions for a droplet in the symmetric T-junction^56^. The viscosity ratio of the droplet to the carrier fluid is 0.1 and the diagram is obtained for various droplet lengths and the capillary numbers *Ca* = *μu/σ* with *µ* and *u* as the continuous phase viscosity and velocity, respectively. As depicted in Fig. 1(a) a droplet with the length of *l*_0_ is moving in a microchannel with width *w* and passes through a symmetric T-junction. Whether the droplet breaks up or not depends on the capillary number (*Ca*) and its dimensionless length (*l*_0_/*w*).

Figure 1(b) shows that our model is in an excellent agreement with the results of Afkhami *et al*.^56^. In fact, all the data points obtained by our model are the same as their results. The solid line *l*_0_/*w* ≈ $0.98{(Ca)}^{-0.21}$ is the result of a theoretical analysis using the thin film approximation.

#### Non-newtonian

**Figure 2:**
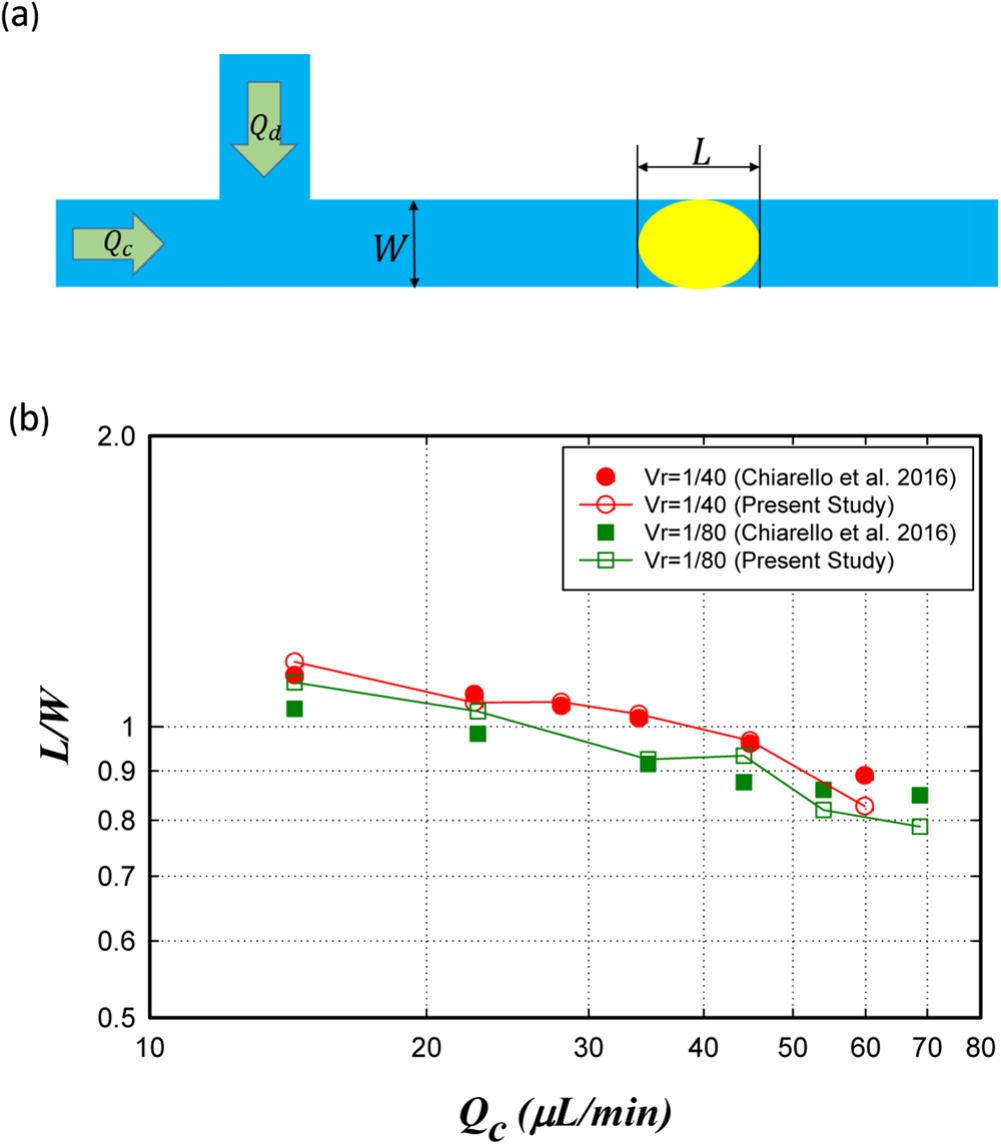
(**a**) The schematic geometry of the system considered for the droplet formation. (**b**) The droplet length as a function of the main channel volume flow rate $({Q}_{c})$ for two volume flow rate ratios $({V}_{r}={Q}_{d}/{Q}_{c})$. The results are in close agreement with the experimental results of Chiarello *et al*. work^57^.

For further verification of our study, we apply our model to a non-Newtonian system that is extensively used for the droplet generation. Chiarello *et al*. have studied the effect of flow rate on the droplet length experimentally^57^. The droplet is soybean oil in a shear thinning Xanthan solution. The carrier fluid is a non-Newtonian power-law fluid with $K=32.6$ mPa s^n^, $n=0.589$ and. mN/m and the dispersed fluid has a viscosity of 49.1 mPa s The microchannel length is 2500 µm and the simulation was conducted with 3000 grids. Figure 2(a) shows the schematic geometry of the considered system. In order to verify the model, the droplet formation in the system and the length of the generated droplet was investigated. Figure 2(b) displays the length of the droplet as a function of the main channel flow rate (${Q}_{c}$) for different flow rate ratios (Q_d_/Q_c_). As it is evident, there is a slight difference between the experimental data and our numerical results. The average and the maximum relative errors are 3.81% and 7.08%, respectively.

### The system under study

**Figure 3:**
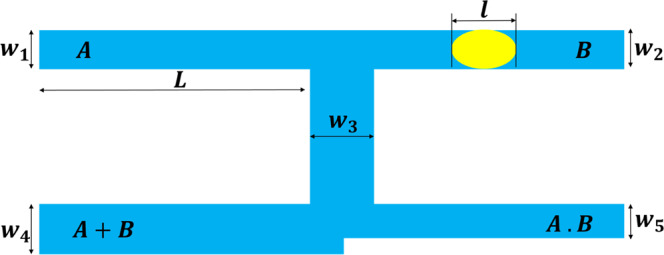
A schematic representation of the considered logic gate in the study and its geometric parameters.

A modified version of the system proposed by Prakash and Gershenfeld is used to study the effects of non-Newtonian fluid on the logic gate operation^22^. Biral *et al*. have also used this geometry in their investigation^58^. Figure 3 shows the geometry of the present logic gate. The channels *A* and *B* are the inlet channels; the channel *A* + *B* is a channel with a larger width and less hydrodynamic resistance (R_4_) and the channel *A.B* is a channel with less width and larger hydrodynamic resistance (R_5_). Therefore, a droplet coming from *A* or *B* will choose the *A* + *B* branch due to its higher flow rate and less hydrodynamic resistance. Thus, we call this state “OR” state. When two droplets are present in the channels *A* and *B*, they will choose both the *A* + *B* and *A.B* branches because when the tip of the droplet enters the *A* + *B* branch it blocks the channel and the hydrodynamic resistance of the branch increases; thus, the rear droplet switches to the *A.B* branch. This is called “AND” state. The geometrical specifications are presented in Table 1.

**Table 1:** The size of the geometrical parameters used in the logic gate.

Stable g	Size (µm)
*W*_1_	50
*W*_2_	50
*W*_3_	70
*W*_4_	50–80
*W*_5_	40
*L*	500

In summary, in order to operate AND state both the inlet channels should contain a droplet and in order to operate OR state at least one droplet should be present in one of the inlet channels.

In the simulation, the inlet boundary condition is constant velocity and the outlet boundary condition is constant pressure. For the walls, the no-slip boundary condition was considered. Since the dimensions of this problem are micrometer size, and the slip length in liquid is in the order of nanometer, so we can use no-slip boundary condition^59–61^.

**Figure 4:**
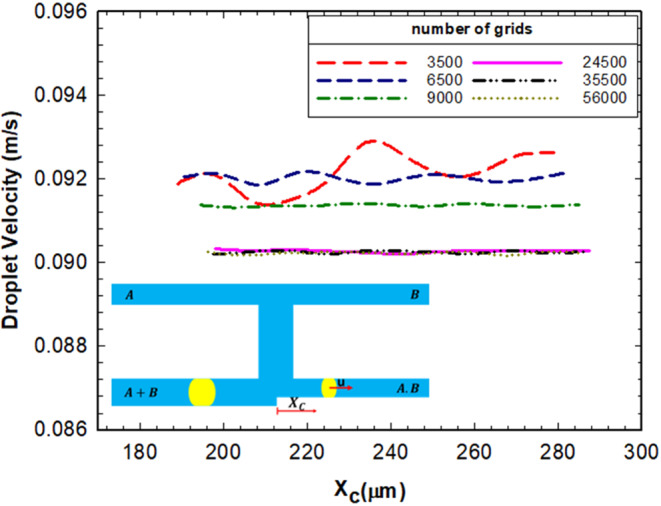
The droplet velocity (u) in the logic gate versus the microchannel axis for different number of grids. Xc is the distance between the center of mass of the droplet and the exit junction.

In order to determine the appropriate number of grids in the study, a grid independence examination was performed. Various number of grids from 3500 to 56000 were considered and the breakup percentage and the droplet velocity (Fig. 4) of the right droplet in AND state were determined and compared. It was realized that the mesh with 24500 grids may be considered as the most efficient one due to its less error (0.55%) and the computational cost. Thus, this mesh was selected for the whole study.

Due to the mesh independency calculations, 24500 grids and a time step of ${10}^{-5}$ sec were considered for the simulations. We investigate how Newtonian and non-Newtonian fluid behavior may affect the logic gates operation. There are some important parameters such as the capillary number, non-Newtonian parameters (*n* and *K* for power-law fluids), the droplet length, and the geometry of microchannel. With the studies on the logic gate, it can be concluded that the following dimensionless parameters are affecting the logic gate operation: ${w}_{4}/{w}_{1}$ (dimensionless channel *A* + *B* width), $l/{w}_{1}$ (dimensionless droplet length), *Ca* = *μu/σ* (the capillary number, where *µ* and *u* represent the continuous phase viscosity and velocity, respectively), and *n* (power-law index). One of the advantages of using dimensionless number is studying the system thoroughly only by changing the parameters that are more appropriate. In the following sections, the effect of each parameter will be investigated.

A non-Newtonian power-law fluid as the dispersed phase and a Newtonian fluid as the continuous phase were considered. The fluids properties are presented in Table 2. The capillary number was changed from 10^−4^ to 0.5.

**Table 2:** The fluid properties used in the study.

Fluid	$\rho$(kg/m^3^)	$\mu$(mPa.s)	$K$ (mPa.s^n^)	$n$	$\sigma$(N/m)
Continuous phase	960	49.1	—	—	0.02
Dispersed phase	1000	—	32.6	0.7–1.3	

### The effects of the power-law index and the bubble/droplet length

**Figure 5:**
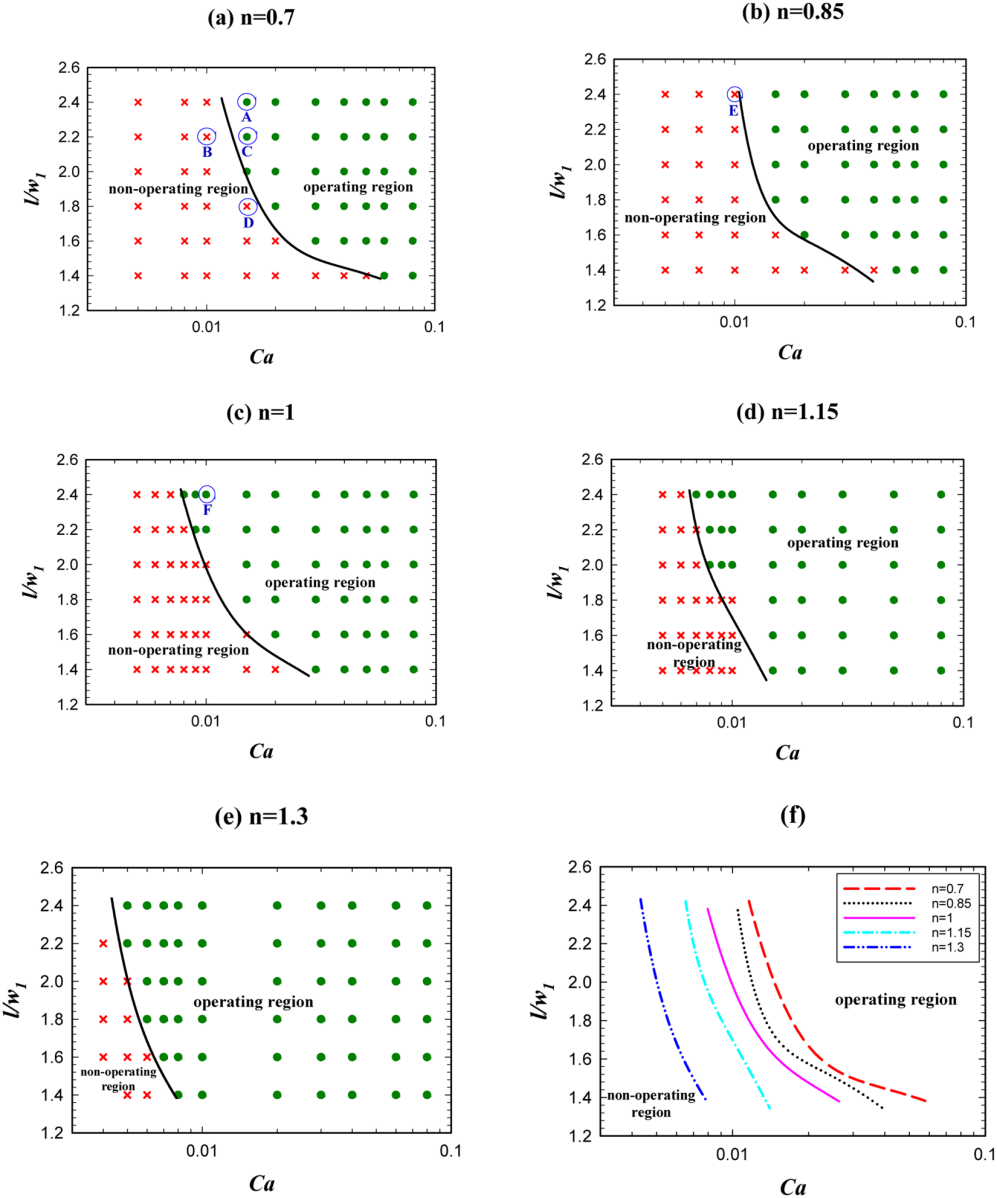
The droplet length effect on AND state for *w*_4_/*w*_1 _= 1.2 and different power-law indexes. (**a**) *n* = 0.7, (**b**) *n* = 0.85, (**c**) *n* = 1, (**d**) *n* = 1.15, (**e**) *n* = 1.3. (**f**) A comparison between the operating and non-operating regions for different power-law indexes.

The droplet length plays an important role in the logic gate operation. A given logic gate may have a proper function in a specified droplet length and an improper function in another droplet length^22^. Thus, investigating the effect of droplet length is an important step in the logic gate design. Figure 5 illustrates the effect of non-dimensional droplet length on AND state of the logic gate in terms of the capillary number for different power-law indexes (*n*) from 0.7 to 1.3. The figure indicates that by increasing the droplet length, the operating region for AND state increases and the droplets with shorter lengths lead to improper operation of AND state. From “the operating region” we mean the conditions that by considering them the system will be in a certain hydrodynamic state in terms of pressure, velocity and vorticity and in this state the droplets may choose one of the outlet branches or may breakup and goes to both the outlet branches. This specifies wheather the AND/OR state works or not.

In AND state, there is one droplet in each inlet channel (A and B). The droplets entered into the system coalescence in the middle channel (w_3_) and form a new droplet. If the droplet breaks up the generated droplets appear in both the exit branches (*A* + *B*, A.B); thus, AND state occurs.

**Figure 6:**
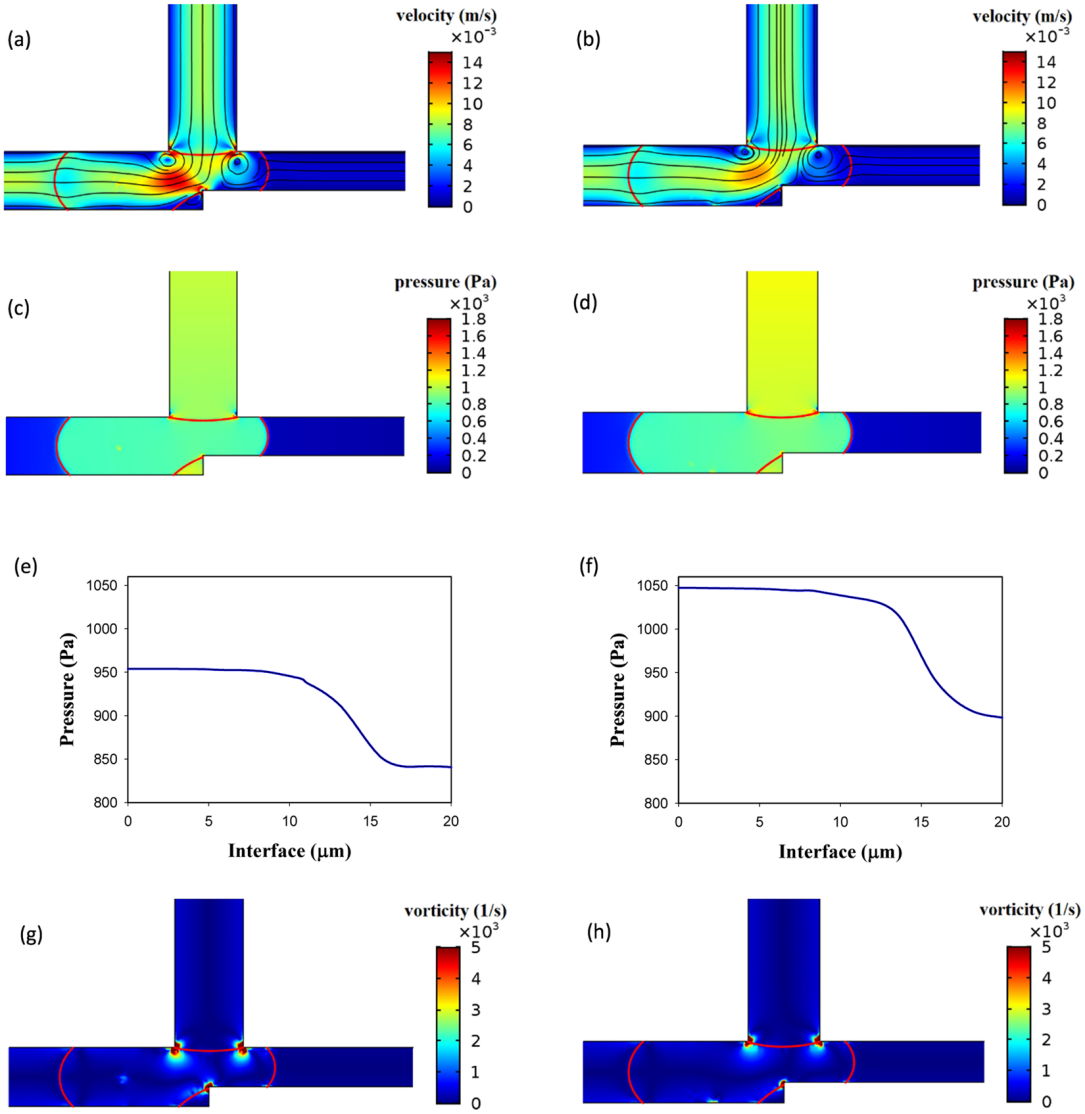
Velocity, pressure and vorticity distributions for a droplet in the entrance of the exit junction in AND state for *w*_4_/*w*_1 _= 1.2, *l*/*w*_1 _= 2.4 and *Ca* = 0.01. The left parts are for *n* = 0.85 in which the droplet does not break up (point E of Fig. 5) and the right parts are for *n* = 1 in which the droplet breaks up (point F of Fig. 5). (**a**,**b**) The velocity distribution (m/s). (**c**,**d**) The pressure distribution (Pa). (**e**,**f**) The pressure drop across the interface. (**g**,**h**) The vorticity distribution (1/s).

Figure 5(f) demonstrates the power-law index influence. As is obvious the operating region increases by increasing *n* from 0.7 to 1.3. By increasing *n*, the viscosity increases and this causes the velocity gradient to be smaller in the droplet. This reduces vortex production and lowers the resistance to deformation and, therefore, the droplet breaks up easily^62^. The velocity, the pressure, and the vorticity of the droplet in the entrance of the exit junction for two different *n* are shown in Fig. 6. The geometry, the droplet length, and the capillary number are the same (*w*_4_/*w*_1 _= 1.2, l/*w*_1 _= 2.4, and Ca = 0.01). AND state in n = 1 (point F of Fig. 5) operates but in n = 0.85 (point E of Fig. 5) does not work. In n = 1 the viscosity is higher and as Fig. 6 illustrates it produces less velocity gradient and vorticity and, thus, the droplet deformation increases. On the other hand, pressure difference across the droplet interface in n = 1 (point F of Fig. 5) and n = 0.85 (point E of Fig. 5) is 149 and 113 Pa, respectively. Therefore, in n = 1 the droplet breakup becomes easier and this facilitates AND state of the logic gate. As can be seen in Fig. 6 the flow field and the pressure change drastically in the droplet breakup region. These will affect the junction area even a while after the breakup. In OR state, the droplet initially is driven to the outlet with less resistance. However, the next droplet may move to the other outlet if the distance between the droplets is not enough. Therefore, the distance between the droplets in the microfluidic systems should be large enough such that the junction area and the outlet channels can recover itself and achieve the same conditions as those experienced by the previous droplet.

**Figure 7:**
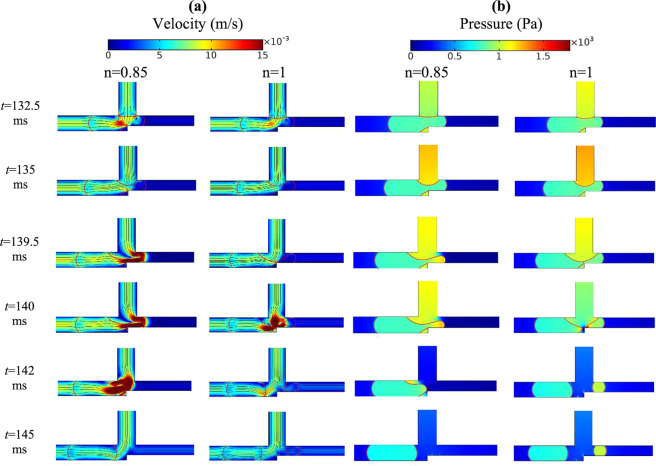
Droplet behavior in AND state in non-breakup (point E of Fig. 5) and breakup (point F of Fig. 5) for *w*_4_/*w*_1 _= 1.2, *l*/*w*_1 _= 2.4 and *Ca* = 0.01, (**a**) evolution of velocity distribution for *n* = 0.85 (non-breakup) and *n* = 1 (breakup), and (**b**) evolution of pressure distribution for *n* = 0.85 (non-breakup) and *n* = 1 (breakup).

Figure 7 shows the snapshots of droplet breakup process as a function of time before and after breakup. The time evolution of the velocity and pressure field for AND state is shown for the non-breakup (point E in Fig. 5) and breakup (point F in Fig. 5). At time *t* = 132.5 ms the merged droplet in the middle channel completely enters the outlet channels. When the droplet with n = 0.85 (point E) enters the bifurcation completely, the pressure behind the droplet tends to squeeze the droplet. However, the pressure and shear force are small compared to the interfacial tension; thus, the deformation rate is not adequate for breaking the droplet. At *t* = 139.5 ms, most of the continuous fluid flows between the right corner of the bifurcation and the droplet. With the passage of time the flow rate increases and this pushes the droplet to pass through the left branch without breakup.

For the droplet with n = 1 (point F) the continuous phase fluid squeezes the middle part of droplet and the neck thickness decreases with time. Then, the droplet breaks up into two droplets. It shows that at *t* = 140 ms the droplet starts to breakup.

**Figure 8:**
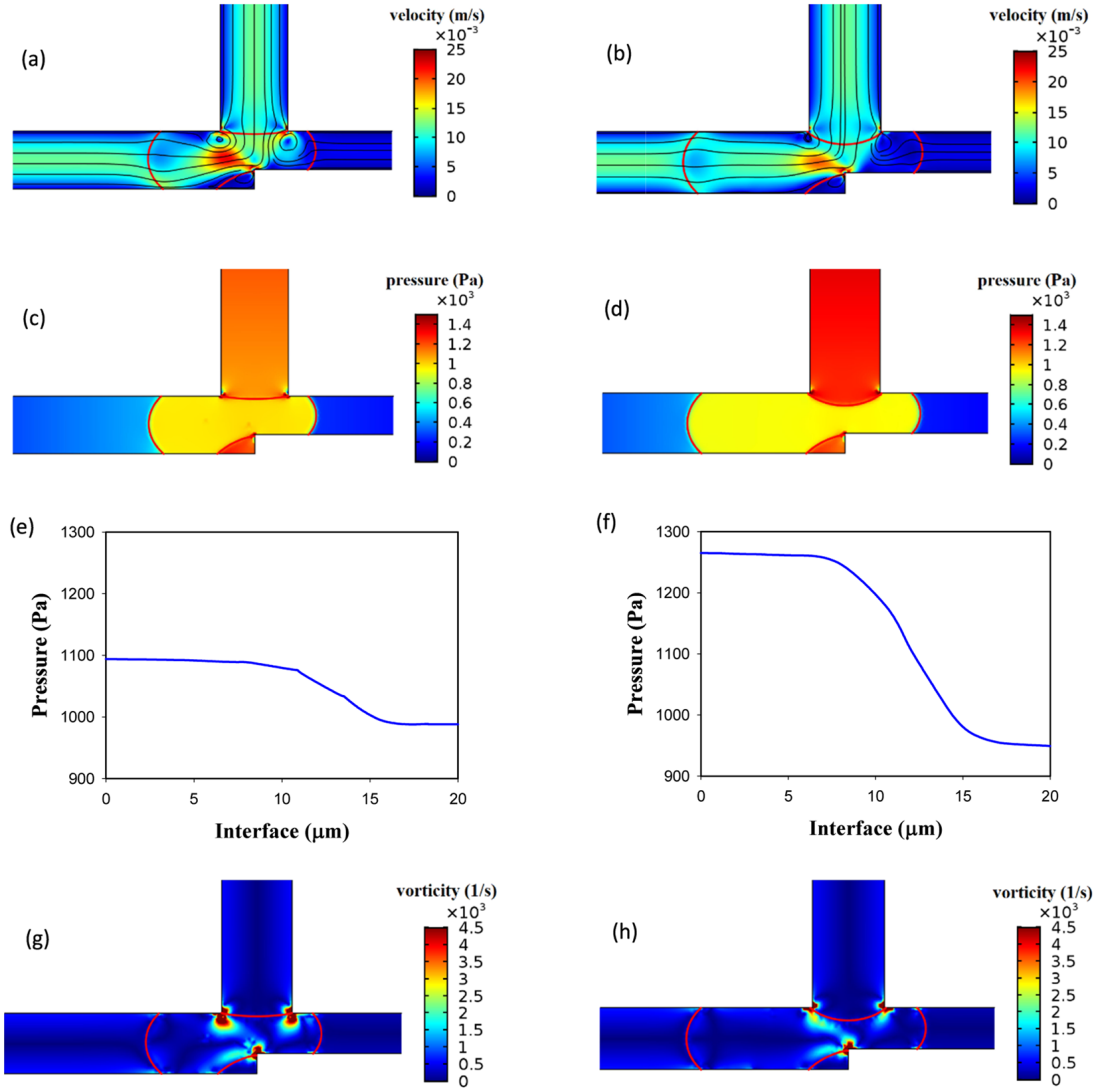
The velocity and the pressure distributions inside a droplet in the entrance of the exit junction in AND state for *w*_4_/*w*_1 _= 1.2, *n* = 0.7 and *Ca* = 0.015. The left parts are for *l*/*w*_1 _= 1.8 in which the droplet does not break up (point D of Fig. 5) and the right parts are for l/*w*_1 _= 2.4 in which the droplet breaks up (point A of Fig. 5). (**a**,**b**) The velocity distribution (m/s). (**c**,**d**) The pressure distribution (Pa). (**e**,**f**) The pressure drop across the interface (Pa). (**g**,**h**) The vorticity distribution (1/s).

As Fig. 8 shows a longer droplet breaks up in the exit junction and, thus, leads to the true AND state. By displaying velocity, pressure and vorticity distributions (points A and D in Fig. 5) the figure explains why the longer droplets assist AND state. The velocity inside the longer droplet (point A) becomes more uniform and the velocity gradient decreases. This limits the vortex creation and leads to weaker vorticities. In addition, the pressure drop across the droplet interface in l/*w*_1 _= 2.4 (point A) and l/*w*_1 _= 1.8 (point D) is 315 and 50 Pa, respectively. All these factors decrease the droplet deformation resistance and the droplet may break up more easily. The snapshots of droplet behavior before and after breakup have been reported in the supplementary information. As is evident in Figs. 6 and 8 the pressure drop in the breakup region of the system is very large and it can be stated that most of the pressure drop occurs in this region. A larger pressure gradient means a smaller time for the breakup. Thus, increasing the capillary number reduces the breakup time.

**Figure 9:**
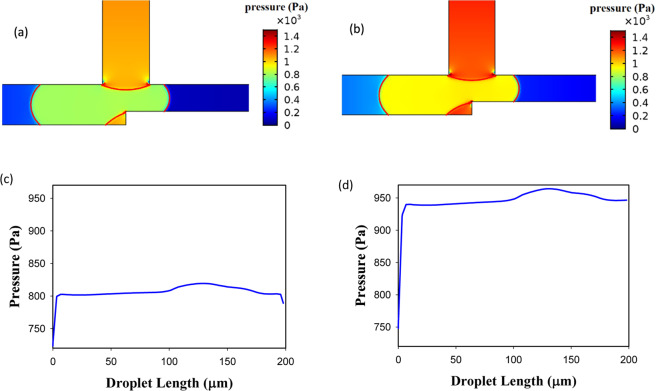
The pressure distributions inside a droplet in the entrance of the exit junction in AND state for *w*_4_/*w*_1 _= 1.2, *l*/*w*_1 _= 2.2 and *n* = 0.7. The left parts are for *Ca* = 0.01 in which the droplet does not break up (point B of Fig. 5) and the right parts are for *Ca* = 0.015 in which the droplet breaks up (point C of Fig. 5). (**a**,**b**) The pressure distribution (m/s). (**c**,**d**) The pressure differences along the droplet (Pa).

The effect of *Ca* in working of AND state of the logic gate has been illustrated in Fig. 9. As can be seen, the gate with a larger *Ca* results in the breakup of the droplet (point C of Fig. 5). The relation between *Ca* number and the breakup has been discussed in many studies^63^. Here, we analyze it by using the pressure distribution along the droplet length. As Fig. 9 shows the pressure differences between the front and the rear of the droplet in *Ca* = 0.01 (point B of Fig. 5) and *Ca* = 0.015 (point C of Fig. 5) is 65 Pa and 200 pa, respectively. Thus, the droplet in *Ca* = 0.015 has a better tendency to break up because of the greater pressure differences. Also, the pressure differences between the front and the center of the droplet and the rear and the center of the droplet are equal to 32 Pa and 97 Pa for *Ca* = 0.01 (point B of Fig. 5) and 19 Pa and 219 Pa for *Ca* = 0.015 (point C of Fig. 5), respectively. The pressure differences in *Ca* = 0.015 is larger and the droplet breakup occurs; AND state is working.

In the previous sections, we studied the effects of droplet length and the power-law index on AND state of the logic gate. In the following section, we will investigate these two parameters on OR state of the logic gate.

**Figure 10:**
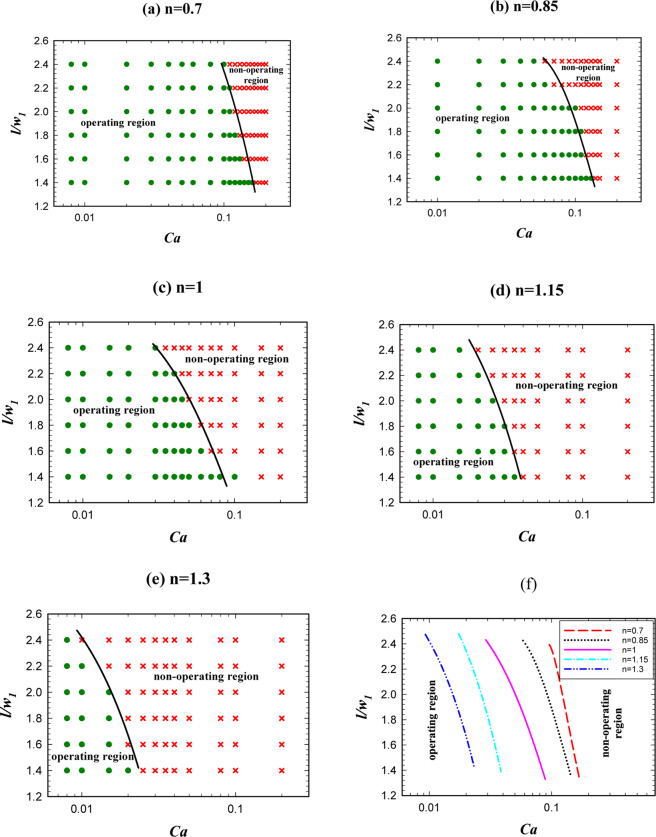
The droplet length effect on the logic gate operation, phase diagram of OR state for different power-law indexes (*w*_4_/*w*_1 _= 1.2). (**a**) *n* = 0.7, (**b**) *n* = 0.85, (**c**) *n* = 1, (**d**) *n* = 1.15, (**e**) *n* = 1.3, (**f**) A comparison between the operating and non-operating regions for different power-law indexes.

As is indicated in Fig. 10, unlike AND state increasing the droplet length, will decrease the operating domain of OR state. This is due to the fact that AND/OR states have two different mechanisms. In AND state two different droplets enter the system from the inlet channels and then they merge in the middle channel and produce a larger droplet. In order to have an AND state the produced droplet should be broken up into two parts in the exit branch. However, in OR state, there is only one droplet in channel *A* or *B*, and it is expected that this droplet passes through the outlet channel with less resistance (*A* + *B*). Thus, the determinative event occurs in the exit junction where the droplet may break up or not. If in AND state the droplet breaks up and in OR state it does not break up and passes through the channel with the lower resistance, the true function of the logic gate is achieved.

With the above explanations, it is obvious that the operating modes of AND/OR states are the opposite of each other. Therefore, by increasing the droplet length, the possibility of droplet breakup increases and the logic gate will no longer have a true function in OR state. In order to avoid prolonging this part, we neglect contours of OR state. The reason for breaking or non-breaking of a droplet is the same as discussed previously. The velocity and pressure contours before and after breakup are available in the supplementary information. Figure 10(f) compares the power-law indexes effect on the operating region of OR state. It is shown that unlike AND state, in OR state by increasing the index from 0.7 to 1.3 the operating region decreases. The reason is due to the reverse function of AND/OR states as discussed above. When the power-law index increases, the viscosity increases. Increasing the viscosity results in the velocity gradient and the vorticity decrease. Thus, the deformation of the droplet increases and the droplet may break up. Consequently, OR state may not work. Thus, increasing the power-law index reduces the operating region of the gate in OR state.

When we design a logic gate we should keep in mind that this gate may sometimes operate in AND state and sometimes in OR state, depending on the specific application. Thus, it is inevitable to know the operating region of both states simultaneously.

**Figure 11:**
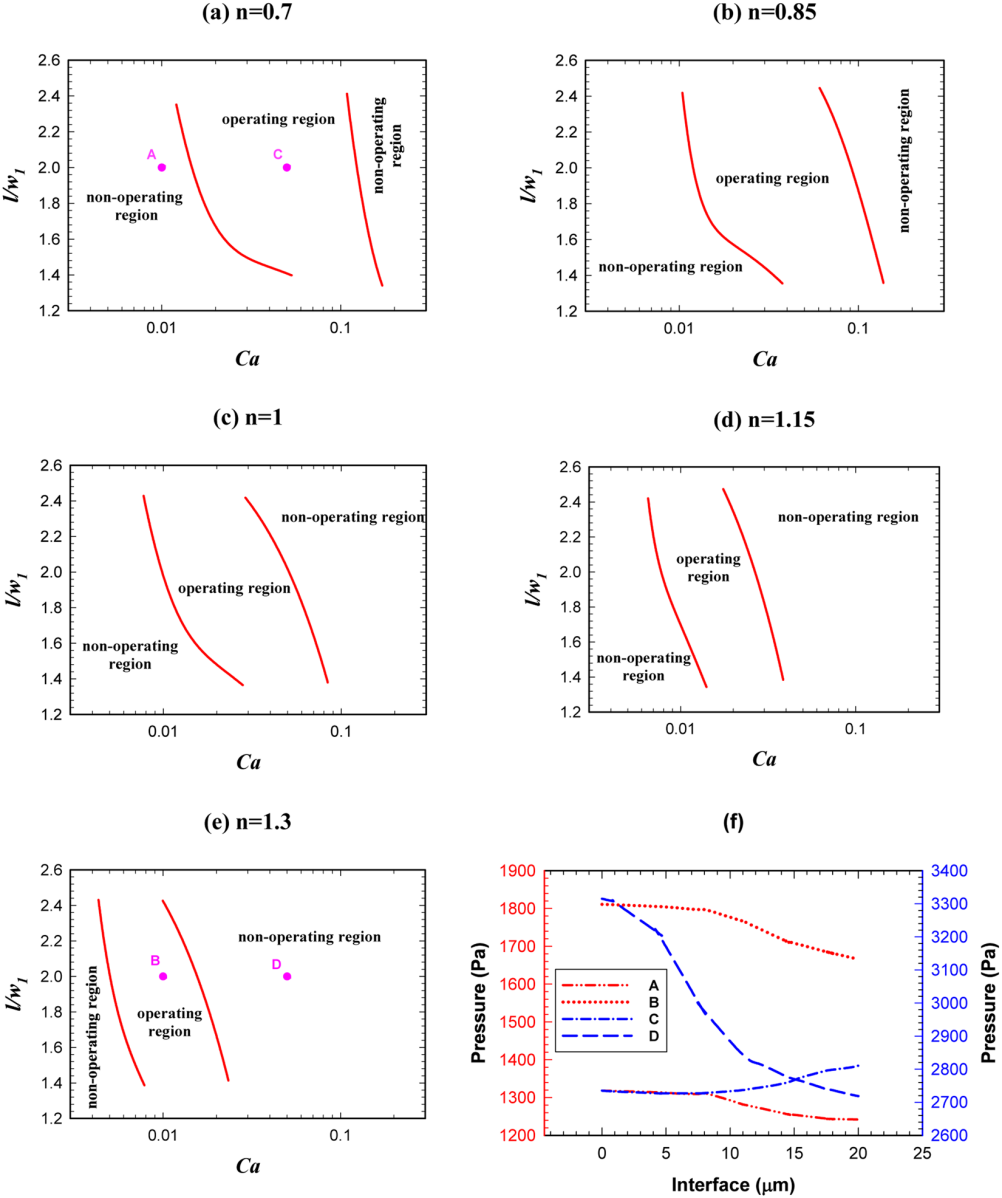
The droplet length effect on the logic gate operation, phase diagram of AND/OR states simultaneously for different power-law indexes (*w*_4_/*w*_1 _= 1.2). (**a**) *n* = 0.7, (**b**) *n* = 0.85, (**c**) *n* = 1, (**d**) *n* = 1.15, (**e**) *n* = 1.3, (**f**) A comparison between the pressure of the operating and non-operating points.

Figure 11 shows the phase diagram in which both AND/OR states will operate. By increasing the power-law index, the operating region of the logic gate becomes narrower and will shift toward the lower capillary numbers. We saw that the phase dividing curve has a large slope in both AND/OR state. The operating region in which both AND/OR states work correctly is presented in Fig. 11 for different power-law indexes. Figure 11(f) explains the reason for decreasing the overall operating region of the logic gate. For four points specified in Fig. 11(a,e) (A, B, C and D) the pressure across the interface of the droplets, when they have completely entered the exit junction, is depicted in Fig. 11(f). Point A (n = 0.7) is in the non-operating region and point B (n = 1.3) is in the operating region. All the parameters except the power-law index are the same in these two points. $l/{w}_{1}$, ${w}_{4}/{w}_{1}$ and $Ca$ are 2, 1.2 and 0.01, respectively. The pressure drop across the interface of the droplet for A and B are 77 and 145 Pa, respectively. On the other hand, as we are in AND state the point B will break up and the gate will work correctly. Points C (n = 0.7) and D (n = 1.3) are also in the operating and non-operating regions, respectively. The pressure across the interface of the droplet in point C increases but, in contrast, it decreases in point D. Because of the pressure change in point D, the droplet breaks up and as we are in OR state the system does not work properly. However, in point C the droplet does not break up and chooses the branch *A* + *B* and the gate works properly. The results indicate a rapid change for the boundary of the regions. Table 3 specifies the range of non-dimensional droplet length in terms of the capillary number for the operating region of the logic gate. The expressions facilitate the use of the results and by using them, it can be easily checked whether a specific point is inside the operating region or not.

**Table 3:** The operating region of a logic gate (range of the droplet length in terms of the capillary number).

*N*	Operating region
0.7	$1.59\exp (\,-\,2.42Ca)+9.78\exp (\,-\,208Ca) < l/{w}_{1} < 1.03\exp (\,-\,0.58Ca)+18.5\exp (\,-\,23.9Ca)$
0.85	$1.85\exp (\,-\,8.17Ca)+156\exp (\,-\,516Ca) < l/{w}_{1} < 143\exp (\,-\,16.3Ca)-141\exp (\,-\,16.9Ca)$
1	$1.76\exp (\,-\,9.12Ca)+10.4\exp (\,-\,332Ca) < l/{w}_{1} < 2034\exp (\,-\,17.38Ca)\,-\,2031\exp (\,-\,17.4Ca)$
1.15	$3\exp (\,-\,57.2Ca)+1424\exp (\,-\,1271Ca) < l/{w}_{1} < 81.8\exp (\,-\,0.64Ca)-78.4\exp (\,-\,0.23Ca)$
1.3	$2.48\exp (\,-\,75.2Ca)+68.9\exp (\,-\,1078Ca) < l/{w}_{1} < 67.1\exp (\,-\,1.14Ca)-63.9\exp (\,-\,0.32Ca)$

### The effects of the geometry

**Figure 12:**
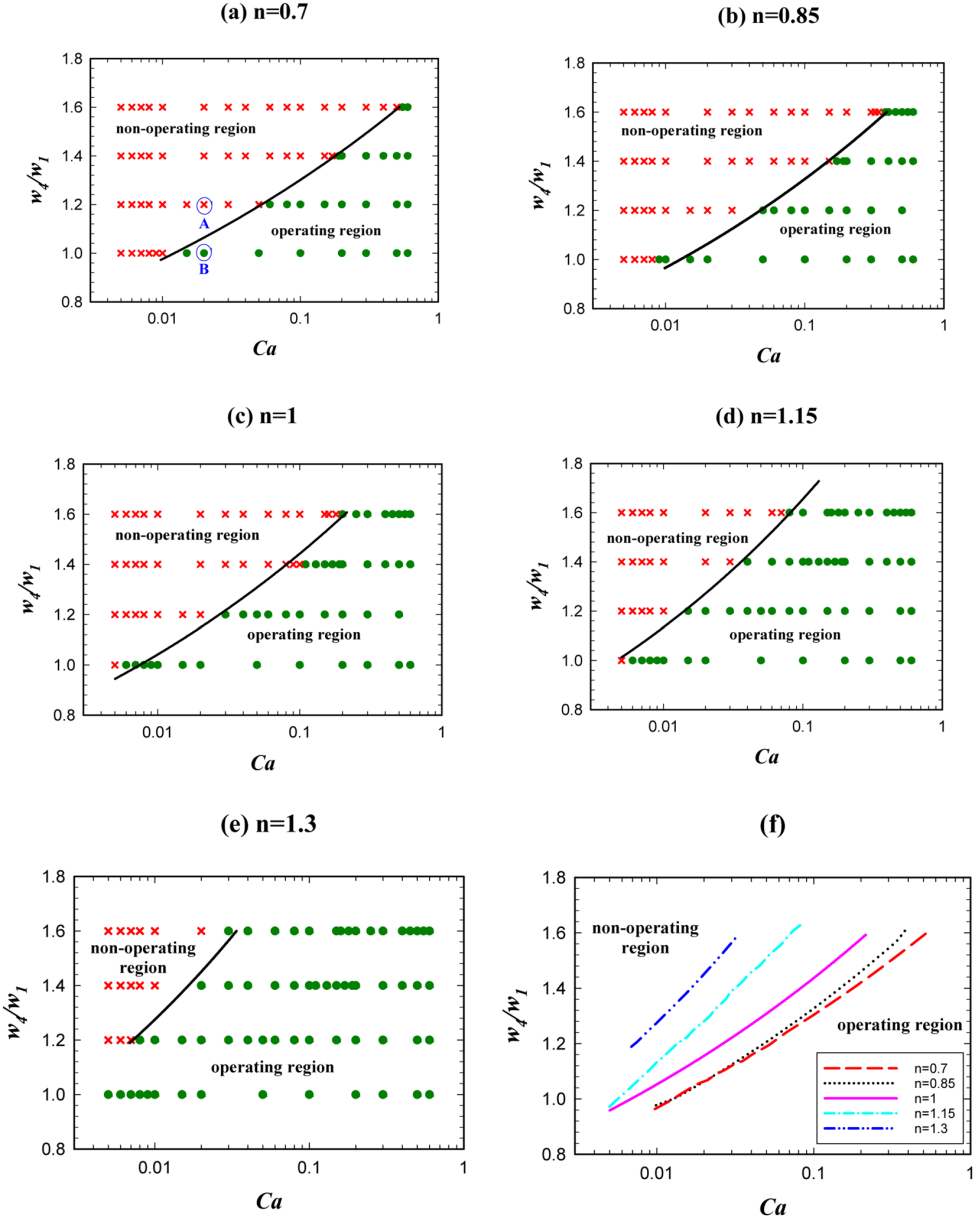
The channel *A* + *B* width effect on AND state of the logic gate for *l*/*w*_1 _= 1.4 and different power-law indexes: (**a**) *n* = 0.7, (**b**) *n* = 0.85, (**c**) *n* = 1, (**d**) *n* = 1.15, (**e**) *n* = 1.3. (**f**) A comparison between the operating and non-operating regions for different power-law indexes.

Another parameter that has an important impact on the operation of the logic gate is the relative hydrodynamic resistance of the two output channels. Here with varying the *A* + *B* channel width (*w*_4_), we change its resistance. With the same length, the wider channel has less hydrodynamic resistance and the droplets prefer to pass through it. Figure 12 shows the effect of non-dimensional channel width on the operation of AND state for different power-law indexes. The non-dimensional channel width was changed from 1 to 1.6. Figure 12(f) compares the operating and non-operating regions for different power-law indexes. As is evident in the figure, the operating region of AND state increases by increasing the power-law index and for the cases with n = 1.3 the largest operating region for AND state is achieved. The reason is that in a certain geometry, fluid, and flow conditions by increasing the power-law index the viscosity of the droplet increases and this leads to a smaller velocity gradient and less vortex. Thus, the resistance to deformation becomes lower and the breakup of the droplet is facilitated.

**Figure 13:**
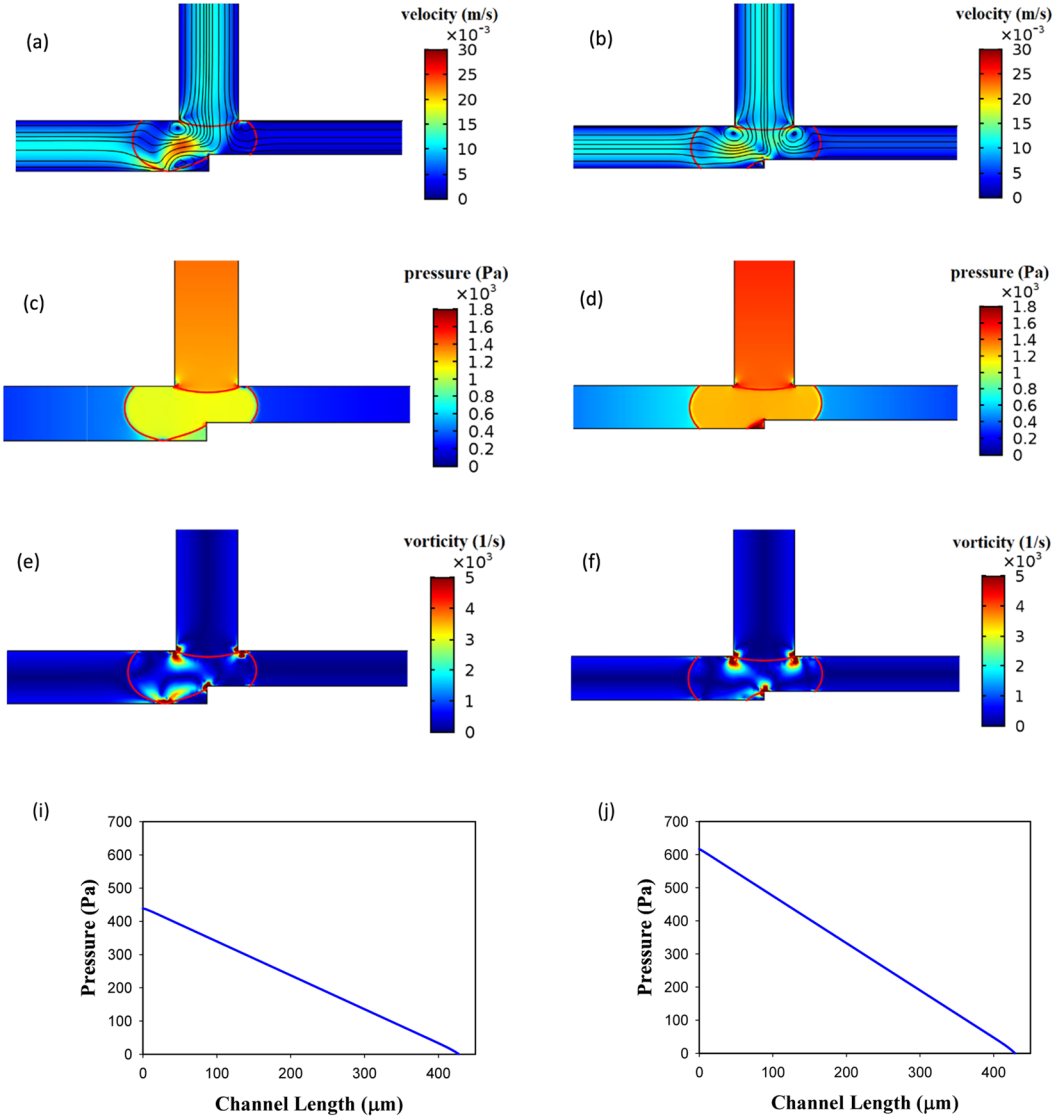
The velocity, the streamline and the vorticity distributions and the pressure drop along the interface of the droplet in the entrance of the exit junction in AND state and for *n* = 0.7, *l*/*w*_1 _= 1.4 and *Ca* = 0.015. The left parts are for *w*_4_/*w*_1 _= 1.2 in which the droplet does not break up (point A) and the right parts are for *w*_4_/*w*_1 _= 1 in which the droplet breaks up (point B). (**a**,**b**) The velocity distributions and streamlines. (**c**,**d**) The pressure distribution. (**e**,**f**) The vorticity distribution. (**g,h**) The pressure drop across the interface. (**i**,**j**)The pressure drop across the channel *A* + *B*.

Figure 12 shows that increasing the channel width (*w*_4_/*w*_1_) decreases the operating region. In this case, the relative resistance of two outlet channels (*w*_4_*/w*_5_) becomes larger and the probability of choosing the channel with the lower resistance by the droplet increases and the droplet breakup may not occur. Therefore, AND state may not operate properly. The detailed phenomenon is shown in Fig. 13. The figure shows the streamlines, velocity and vorticity distributions for two different widths of the channel *A* + *B* for AND state. The left parts are for *w*_4_/*w*_1 _= 1.2 (point A) and the right parts are for *w*_4_/*w*_1 _= 1 (point B). All the other parameters are the same in the parts. The dimensionless droplet length is 1.4, the capillary number is 0.015 and *n* is 0.7. AND state is working for *w*_4_/*w*_1 _= 1 in which the droplet breaks up. As it is obvious, the velocity gradient is bigger in the wider channel (left one) and the vorticity becomes stronger. Thus, the droplets do not tend to deform. The pressure drop across the droplet interface is 150 and 185 Pa for *w*_4_/*w*_1 _= 1 (point B) and *w*_4_/*w*_1 _= 1.2 (point A), respectively. However, the pressure drop along the channel *A* + *B* is 617 and 439 Pa for *w*_4_/*w*_1 _= 1 (point B) and *w*_4_/*w*_1 _= 1.2 (point A), respectively. It means that when the channel becomes wider the pressure drop along it decreases and the droplet prefers to enter to this channel and will not break up. Thus, AND state will no longer work by making the channel wider. Although the pressure drop across the droplet interface in the entrance of the junction is 35 Pa more in the wider channel, it does not lead to the droplet breakup. In the wider channel, the pressure drop along the channel becomes more important than the pressure drop across the interface of the droplet. The pressure drop differences along the channel and across the interface between *w*_4_/*w*_1 _= 1 (point B) and *w*_4_/*w*_1 _= 1.2 (point A) are 178 and 35 Pa, respectively. Therefore, the dominant phenomenon is the pressure drop along the channel and this factor controls the droplet break up process. The more detailed snapshots of the breakup process and the effect of channel width ratio on the droplet breakup percentage in two outlet channels are available in the supplementary information.

**Figure 14:**
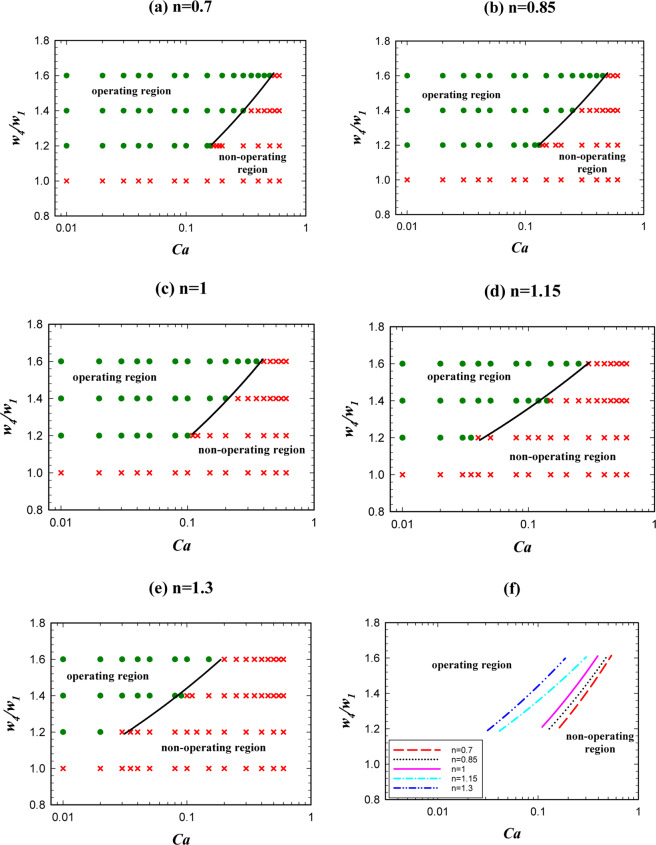
The channel *A* + *B* width effect on OR state of the logic gate for *l*/*w*_1 _= 1.4 and different power-law indexes. (**a**) *n* = 0.7, **(b**) *n* = 0.85, (**c**) *n* = 1, (**d**) *n* = 1.15, (**e**) *n* = 1.3, (**f**) A comparison between the operating and the non-operating regions for different power-law indexes.

Figure 14 shows the effect of the channel width on OR state of the logic gate. Unlike AND state, the operating region of the logic gate increases by increasing the channel *A* + *B* width in the OR state. A general reason is that by increasing the width of the channel *A* + *B* the relative width (*w*_4_*/w*_5_) and the relative resistance (*R*_5_*/R*_4_) of the two channels increase. This means that the difference between the hydrodynamic resistance of the two channels increases and the main inlet flow will pass through the wider channel (*A* + *B*) and the droplet that reaches the exit junction tends to choose this channel. In order to avoid prolonging this part, we do not present the related contours of OR state.

**Figure 15:**
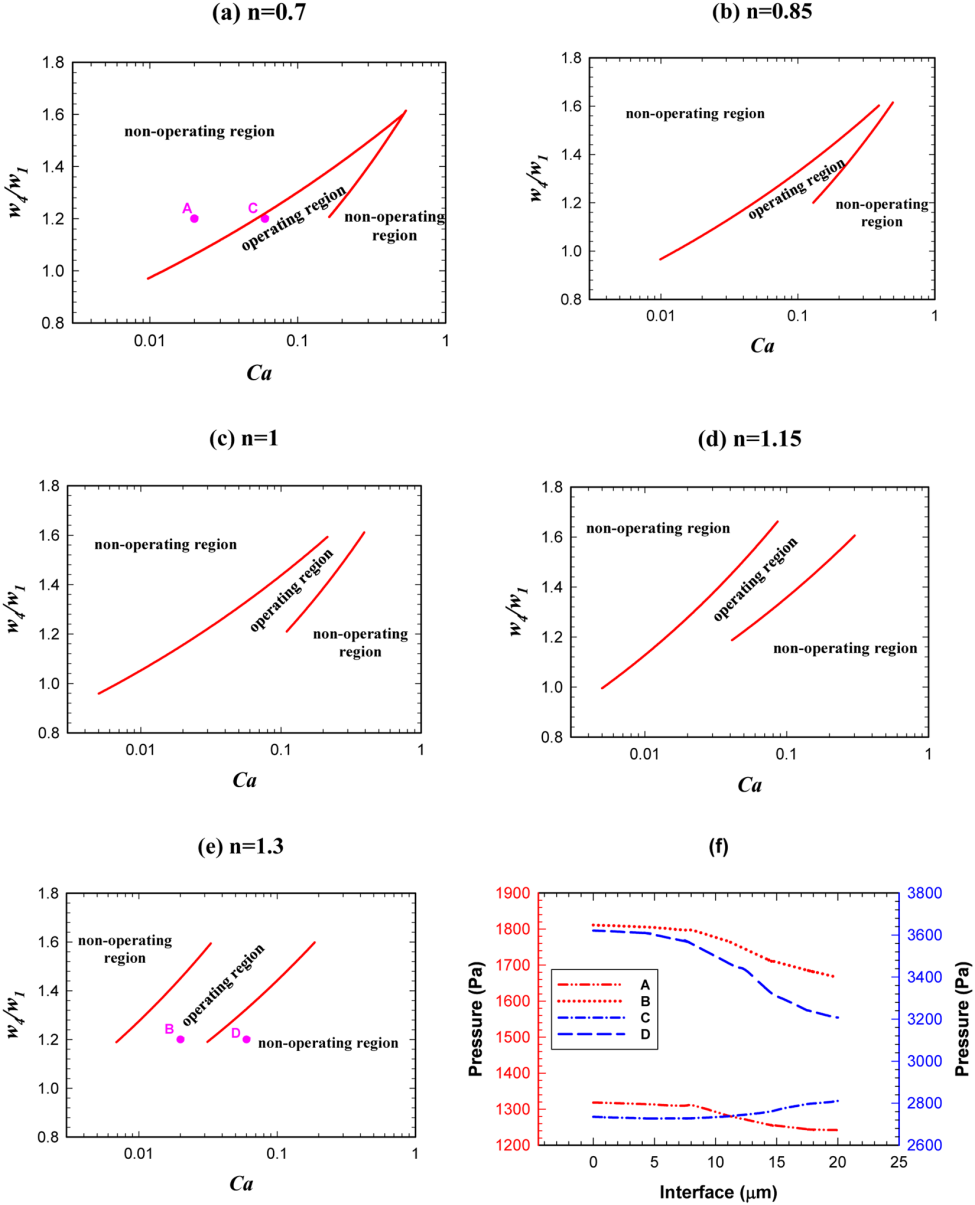
The channel *A* + *B* width effect on the logic gate operation (both the states simultaneously) for *w*_4_/*w*_1 _= 1.2 and different power-law indexes. (**a**) *n* = 0.7, (**b**) *n* = 0.85, (**c**) *n* = 1, (**d**) *n* = 1.15, (**e**) *n* = 1.3, (**f**) A comparison between the pressure of the operating and non-operating points.

The operating regions of AND/OR states have been presented together in Fig. 15 for different power-law indexes. The figure indicates that the operating region increases by increasing the power-law index. The reason is shown briefly in Fig. 15(f). Two points (*A, C*) for n = 0.7 have been compared with the corresponding points (*B, D*) for n = 1.3. Apart from the power-law index, the rest of the parameters are the same for the corresponding points. The pressure drop in point *A* and *B* are 77 and 145 Pa, respectively. As these points are in AND state, the droplet in *B* breaks up and the AND state works. The pressure across the interface of the droplet in the point *C* increases 75 Pa whereas in point *D* decreases 597 Pa. Since these points operate in OR state the droplet in the point *D* will break up and the OR gate will not work. However, the droplet in point *C* does not break up and will choose the branch *A* + *B*; thus, OR state will work. Table 4 specifies the range of non-dimensional channel width in terms of the capillary number for the operating region of the logic gate. The results indicate that the profiles follow a power law distribution.

**Table 4:** The operating region of a logic gate (range of the channel width in terms of the capillary number).

*n*	Operating region
0.7	$1.74C{a}^{0.13} < {w}_{4}/{w}_{1} < 1.88C{a}^{0.24}$
0.85	$1.82C{a}^{0.14} < {w}_{4}/{w}_{1} < 1.89C{a}^{0.22}$
1	$2C{a}^{0.14} < {w}_{4}/{w}_{1} < 1.99C{a}^{0.22}$
1.15	$1.93C{a}^{0.15} < {w}_{4}/{w}_{1} < 2.42C{a}^{0.16}$
1.3	$3C{a}^{0.19} < {w}_{4}/{w}_{1} < 3.01C{a}^{0.19}$

## Conclusion

We have studied new aspects of logic gates and their operating conditions. Moreover, all the previous studies in the field of the microfluidic logic gate had used Newtonian fluids. Since most of the fluids are non-Newtonian, it was very important to study the effect of these types of fluids and their properties in the microfluidic logic gates. It was revealed that there are limitations for a logic gate to work properly. Important factors such as the droplet length, the non-Newtonian properties, the capillary number and the geometry of the logic circuit will affect the operating range of a logic gate. We studied all these parameters and explained how they may affect logic gate operation. Another novelty of our work was studying the non-Newtonian fluids in the logic gate.

It was shown that the mechanism of AND/OR states are opposite each other. Thus, there is a specific operating region that is obtained from the subscription of operating regions of AND/OR states. In AND state, increasing the capillary number, the droplet length and the power-law index, causes an increase in the operating region. In contrast, increasing the width of the branch *A* + *B* decreases the operating region. In OR state increasing the capillary number, the droplet length and the power-law index, results in decreasing the operating region of the gate. while increasing the width of the branch *A* + *B* increases the operating region. Since the operation of a logic gate depends on both AND/OR states, there is a range of physical and geometrical properties in which a logic gate will work properly. We have derived these ranges so that for designing a logic gate, one can refer to these plots to design an optimum gate.

## Supplementary information

Supplementary information.
