# Supplementary material for: Non-Newtonian droplet-based microfluidics logic gates
Source: Sci Rep. 2020 Jun 9;10:9293. doi: 10.1038/s41598-020-66337-7 (PMC7283233; doi:10.1038/s41598-020-66337-7)
Supplement: Supplementary file 1 — Supplementary information. [file 41598_2020_66337_MOESM1_ESM.pdf]

Non-Newtonian droplet-based microfluidics logic gates

Elmira Asghari, Ali Moosavi [a)], Siamak Kazemzadeh Hannani

Center of Excellence in Energy Conversion (CEEC), School of Mechanical Engineering, Sharif University of Technology, Azadi Avenue, P. O. Box 11365-9567, Tehran, Iran

Results and discussion

The effects of the power-law index and the bubble/droplet length

Figure 1 shows the snapshots of droplet breakup process in times before and after breakup for two different droplet lengths l/w_1=1.8 and l/w_1=2.4. The time evolutions of velocity and pressure fields for AND state are shown for the non-breakup (l/w_1=1.8) and breakup (l/w_1=2.4). When the droplet with length l/w_1=1.8 enters the bifurcation completely, the pressure and shear force are small compared to the interfacial tension. Thus, the deformation rate is not adequate for breaking the droplet. At t= 92 ms, most of the continuous fluid flows between the right corner of the bifurcation and the droplet. With the passage of time, the flow rate increases and this pushes the droplet to pass through the left branch without breakup.

For the droplet with l/w_1=2.4 the continuous phase fluid squeezes the middle part of droplet. The neck thickness decreased with time. Then, the droplet breaks up into two droplets. It shows that at t=92 ms the droplet starts to breakup.

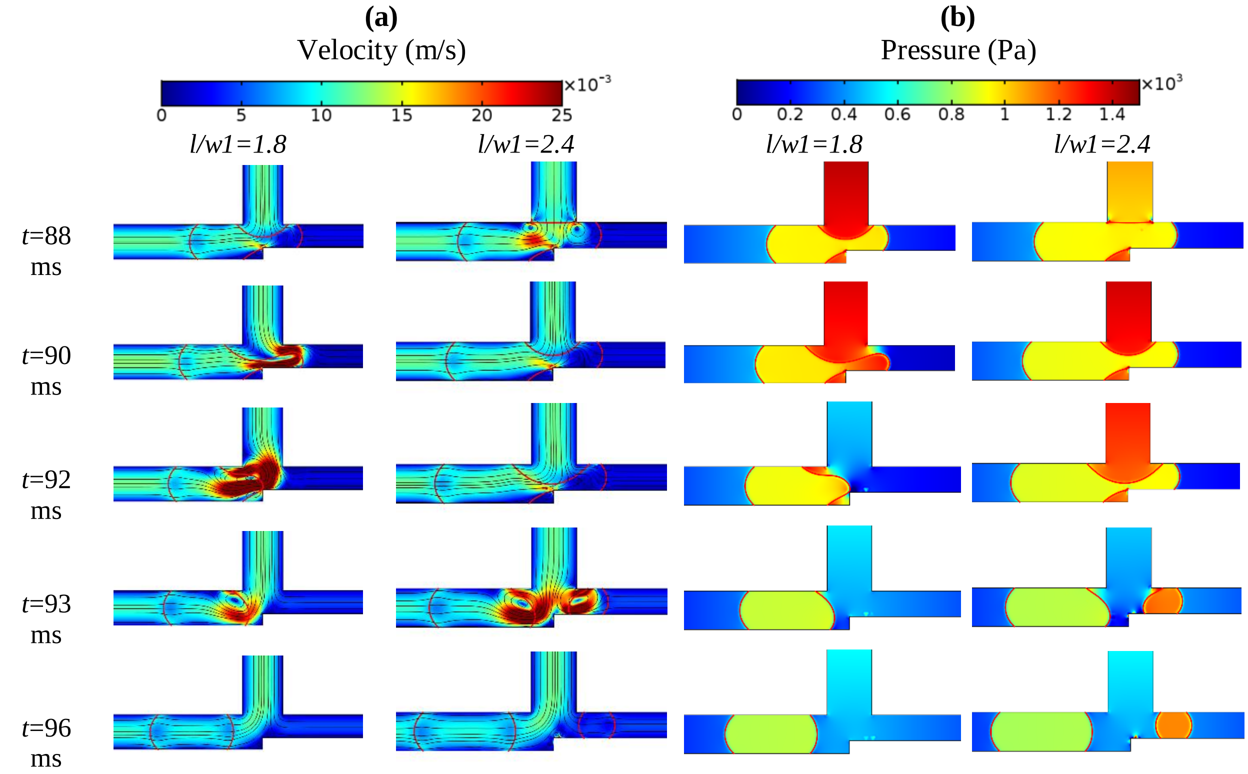

Figure 1. Droplet behavior in the AND state in non-breakup (point D of Fig. 5) and breakup (point A of Fig. 5) for *w*4/*w*1=1.2, *n*=0.7 and *Ca*=0.01, (a) evolution of velocity distribution for l/w1=1.8 (non-breakup) and *l*/*w*1=2.4 (breakup), and (b) evolution of pressure distribution for l/*w*1=1.8 (non-breakup) and l/*w*1=2.4 (breakup).

Figure 2 shows the snapshots of droplet breakup process for different times before and after breakup. The time evolutions of the velocity and pressure fields for OR state are shown for the non-breakup (*n*=1) and breakup (*n*=1.15). At time *t*=64 ms the merged droplet in the middle channel completely enters the outlet channels. When the droplet with *n*=1 enters the bifurcation completely, the pressure behind the droplet tends to squeeze the droplet but the pressure and shear force are small compared to the interfacial tension. Thus, the deformation rate is not adequate for breaking the droplet. At *t*= 66 ms, most of the continuous fluid flows between the right corner of the bifurcation and the droplet. The flow rate gradually increases and this pushes the droplet to pass through the left branch without breakup. Therefore, the OR state is working.

For the droplet with *n*=1.15 the continuous phase fluid squeezes the middle section of droplet. The neck thickness decreases with time. Then, the droplet breaks up into two droplets. It shows that at *t*=70 ms the droplet starts to breakup. Therefore, the OR state is not working.

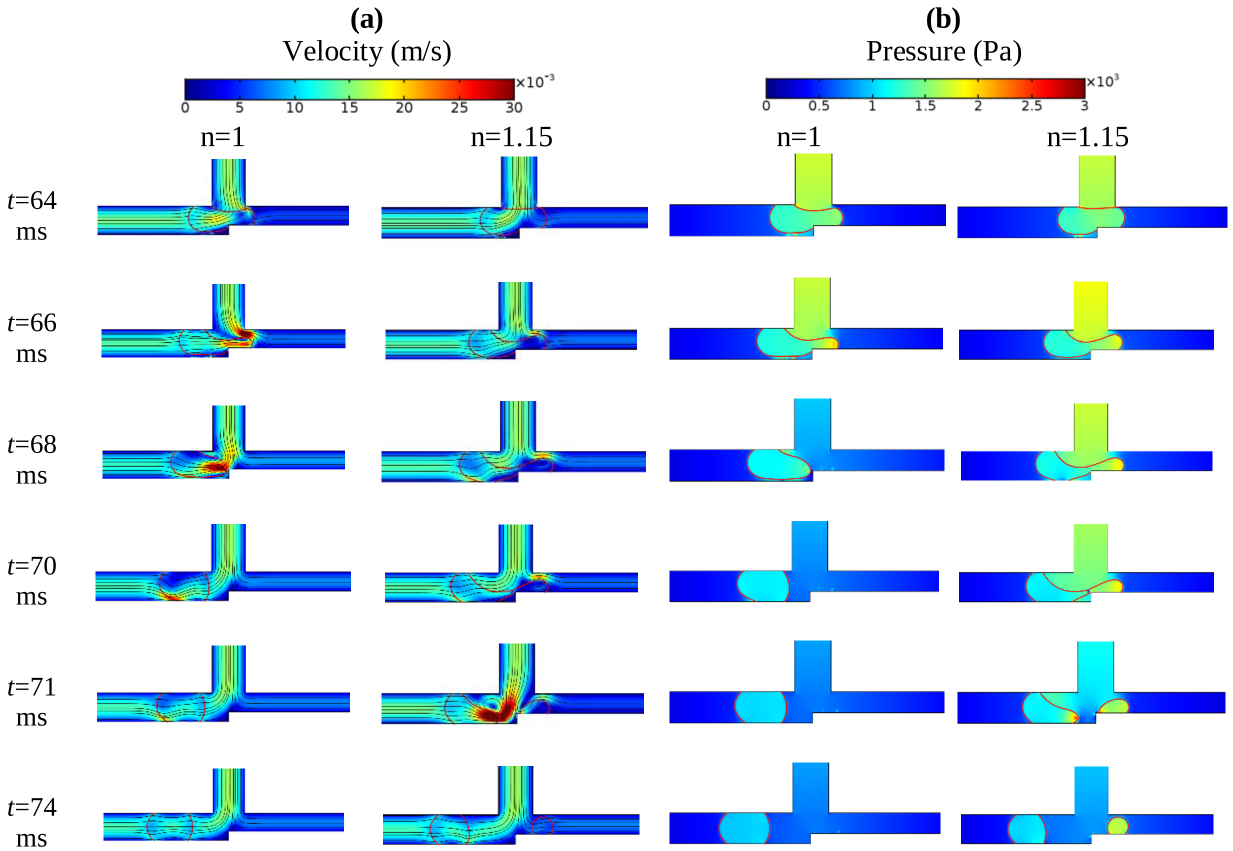

Figure 2- droplet behavior in OR state for w4/w1=1.2, l/w1=2.4 and Ca=0.02, (a) evolution of velocity distribution for n=1 (non-breakup= true OR) and n=1.15 (breakup= false OR), and (b) evolution of pressure distribution for n=1 (non-breakup) and n=1.15 (breakup).

Figure 3 shows the snapshots of droplet breakup process for different times before and after breakup. The time evolutions of velocity and pressure fields for OR state are shown for the non-breakup (*l/w*1=2.2) and breakup (*l/w*1=2.4). At time *t*=130 ms the merged droplet in the middle channel completely enters the outlet channels. When the droplet with *l/w1*=2.2 enters the bifurcation

completely, the pressure behind the droplet tends to squeeze the droplet but the pressure and shear force are small compared to the interfacial tension. Consequently, the deformation rate is not adequate for breaking the droplet. At *t*= 143 ms, most of the continuous fluid flows between the right corner of the bifurcation and the droplet. With the passage of time, the flow rate increases, and this pushes the droplet to pass through the left branch without breakup. Therefore, the OR state is working.

For the droplet with *l/w1*=2.4 the continuous phase fluid squeezes the middle part of the droplet. The neck thickness decreases with time. Then, the droplet breaks up into two droplets. It shows that at *t*=144 ms droplet starts to breakup. Therefore, the OR state is not working.

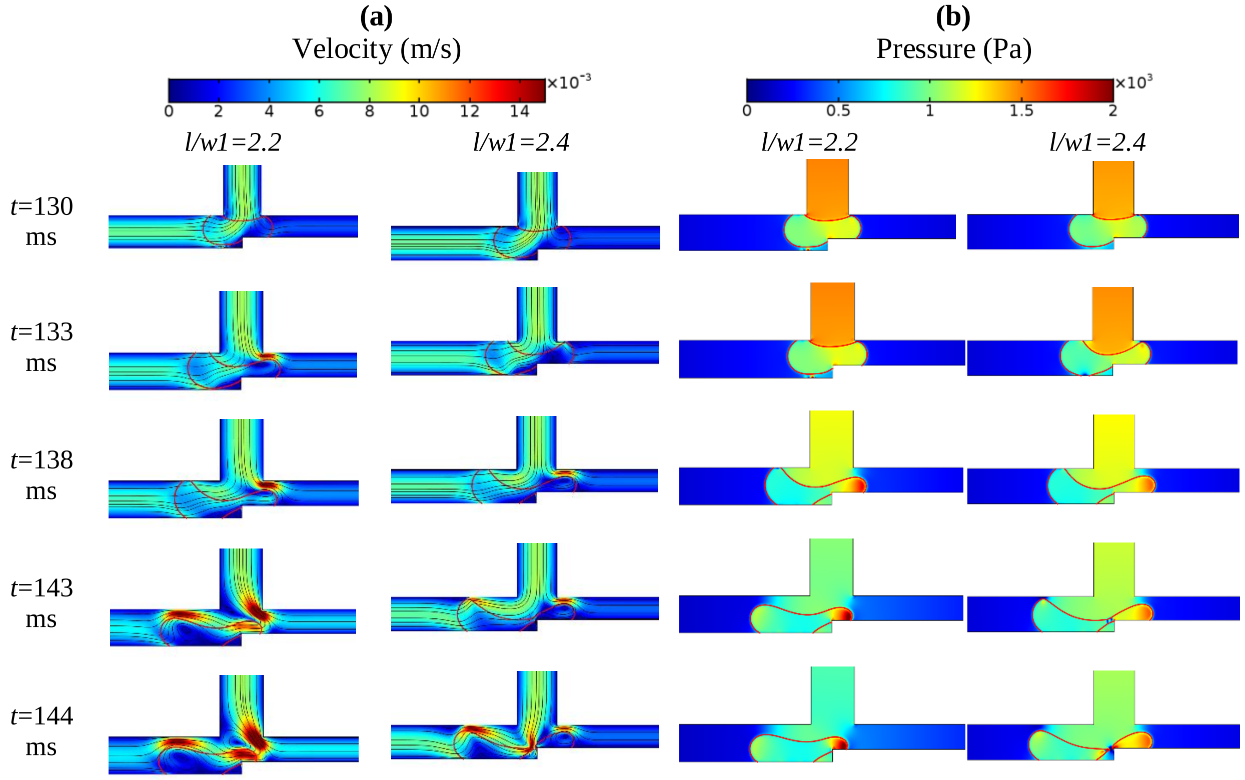

t=148 ms

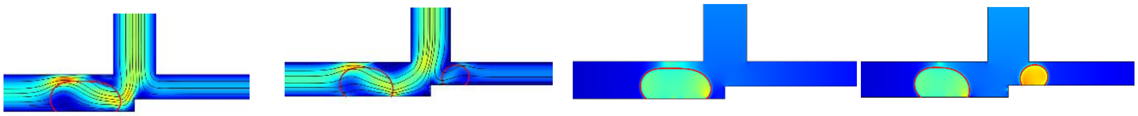

Figure 3. Droplet behavior in OR state for *w*4/*w*1=1.2, *n*=1.3 and *Ca*=0.02, (a) evolution of velocity distribution for *l*/*w*1=2.2 (non-breakup= true OR) and *l*/*w*1=2.4 (breakup= false OR), and (b) evolution of pressure distribution for *l*/*w*1=2.2 (non-breakup) and *l*/*w1*=2.4 (breakup).

The effects of the geometry

Figure 4 shows the snapshots of droplet breakup process for different times before and after breakup. The time evolutions of the velocity and pressure fields for the AND state are shown for the non-breakup (*w*4/*w*1=1.2) and breakup (*w*4/*w*1=1). At time *t*=85 ms the merged droplet in the middle channel completely enters the outlet channels. when the droplet with *w*4/*w*1=1.2 enters the bifurcation completely, the pressure behind the droplet tends to squeeze the droplet. However, the pressure and shear force are small compared to the interfacial tension; thus, the deformation rate is not adequate for breaking the droplet. At *t*= 87 ms, most of the continuous fluid flows between the right corner of the bifurcation and the droplet. With the passage of time, the flow rate increases and this pushes the droplet to pass through the left branch without breakup. Therefore, the AND state is not working.

For the droplet with *w*4/*w*1=1 the continuous phase fluid squeezes the middle section of droplet. The neck thickness decreased as the time goes on. Then the droplet breaks up into two droplets. It shows that at *t*=89 ms droplet starts to breakup. Therefore, the AND state is working.

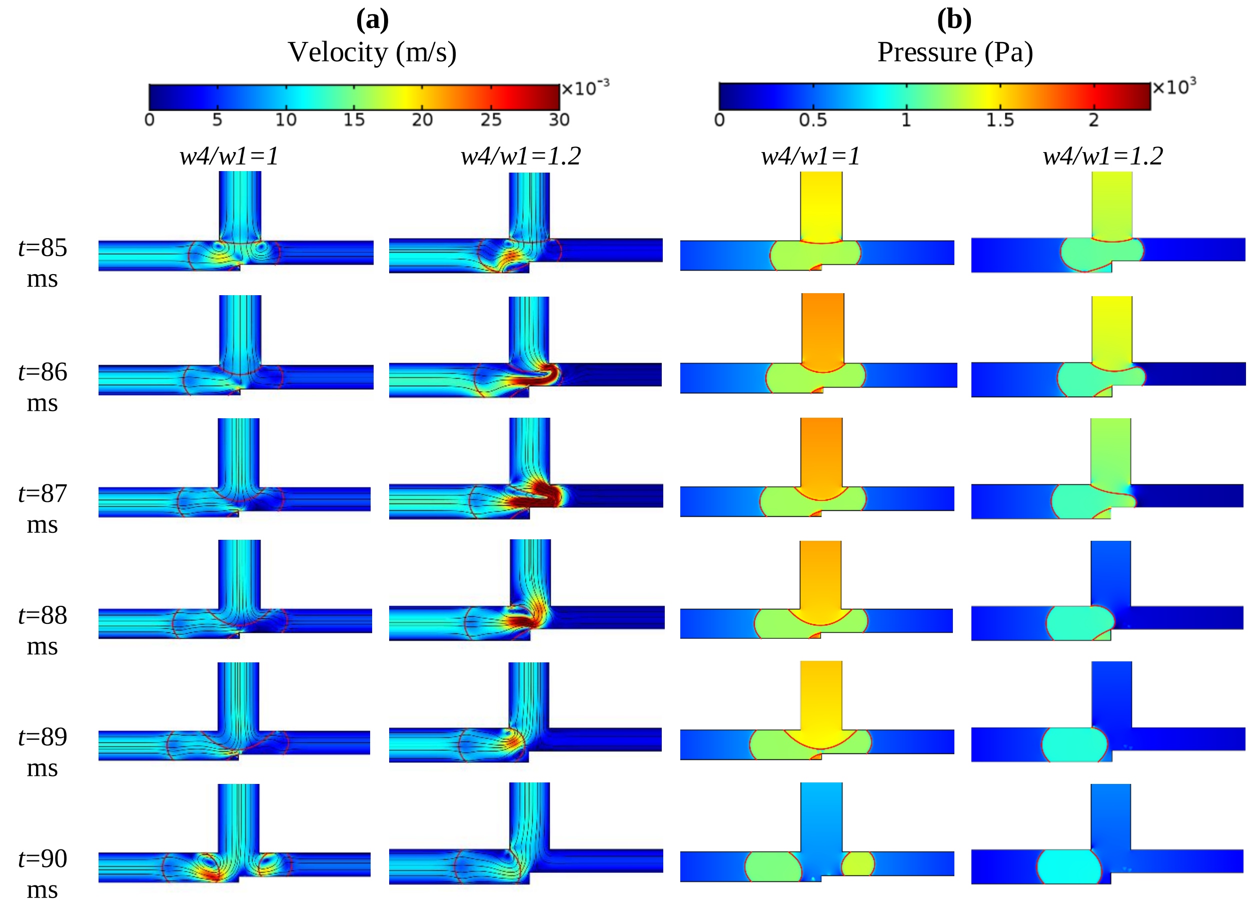

Figure 4. Droplet behavior in AND state in non-breakup (point A of Fig. 12) and breakup (point B of Fig. 12) for $l/w1$=1.4, n=0.7 and Ca=0.015, (a) evolution of velocity distribution for $w4/w1$=1.2 (non-breakup) and $w4/w1$=1 (breakup), and (b) evolution of pressure distribution for $w4/w1$=1.2 (non-breakup) and $w4/w1$=1 (breakup).

Effect of physical parameters on the breakup

According to investigation of Bedram et al. the most important parameter which affect the droplet size, is the channel width ratio (w_4/w_1)[1]. As the droplet is non-Newtoian, we consider the effects of fluid power index (n), too. The results are as below:

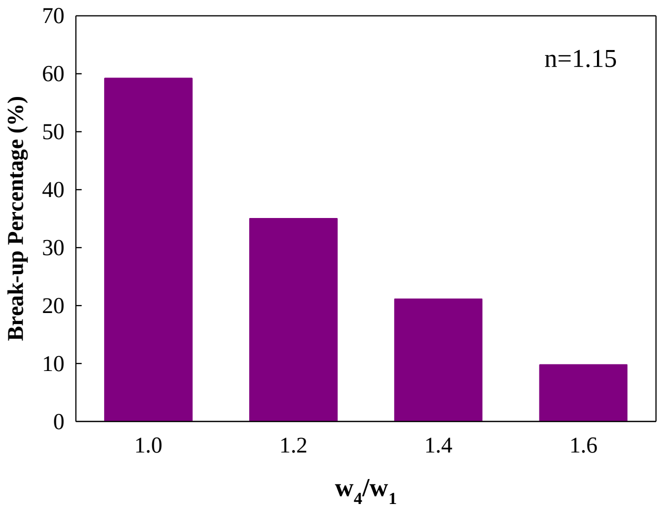

Figure 5. The percentage of volume ratio of daughter droplets after breakup in terms of different channel width ratio ($w4/w1$) for $l/w1$=1.4, n=1.15 and Ca=0.1.

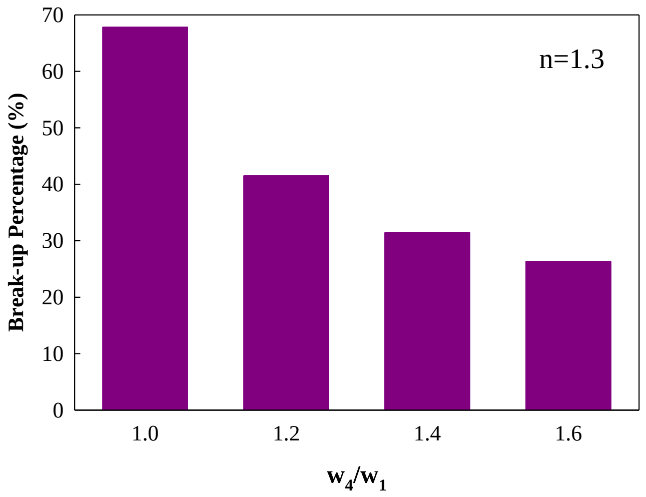

Figure 6. The percentage of the volume ratio of daughter droplets after breakup in terms of different channel width ratio ($w4/w1$) for $l/w1$=1.4, n=1.3 and Ca=0.1.

Breakup percentage is defined as:

$$\text{Breakup percentage} = \frac{\text{droplet volume in the right channel (A.B)}}{\text{droplet volume in the left channel (A + B)}} \times 100$$

Figures 5 and 6 show that the volume of the droplet which that goes to the right channel (A.B) is decreaseing while increasing the channel width ratio (w_4/w_1). Because when the channel width ratio (w_4/w_1) increses, it means that the hydrodynamic resistance of the left channel (A+B)

decreases and more flow rate passes through channel A+B. Thus, more volume of the droplet tends to go to the channel A+B and less to the channel A.B. Consequently, when the droplet breakup occurs, the breakup percentage decreases.

Figures 5 and 6 also show that increasing the fluid power index (*n*), increases the breakup percentage. Because by increasing *n*, the viscosity increases and this causes the velocity gradient to be smaller in the droplet. This reduces vortex production and lowers the resistance to deformation and, therefore, the droplet breaks up faster. It means that when the droplet enters the exit branches, the deformation rate is enough to start the breakup and there is less time for the droplet to go to channel A+B. Thus, the droplet volume ratio increases.

References

1. Bedram, A. & Moosavi, A. Droplet breakup in an asymmetric microfluidic T junction. *European Physical Journal E* **34**, (2011).
